# Are we preparing for the digital healthcare era?

**DOI:** 10.1590/1516-3180.2022.140225112021

**Published:** 2022-01-31

**Authors:** Guilherme Machado Rabello, Paulo Manuel Pêgo-Fernandes, Fabio Biscegli Jatene

**Affiliations:** I Engineer, Escola Politécnica da Universidade de São Paulo. Innovation Manager of InovaInCor, Instituto do Coracao, Hospital das Clinicas HCFMUSP, Faculdade de Medicina, Universidade de Sao Paulo, Sao Paulo, SP, BR.; II MD, PhD. Full Professor, Thoracic Surgery Program, Instituto do Coracao, Hospital das Clinicas HCFMUSP, Faculdade de Medicina, Universidade de Sao Paulo, Sao Paulo, SP, BR; Director, Scientific Department, Associação Paulista de Medicina (APM), São Paulo (SP), Brazil.; III MD, PhD. Full Professor, Cardiovascular Surgery Division, Coordinator of InovaInCor, Instituto do Coracao, Hospital das Clinicas HCFMUSP, Faculdade de Medicina, Universidade de Sao Paulo, Sao Paulo, SP, BR

At a handful of moments over the history of human society, major revolutions have given rise to profound social changes. The creation of the wheel, Gutenberg’s press machine, electricity, the telephone and, more recently, the internet have all changed human history.^1^ Another tipping point was reached in early 2020, with the advent of the severe acute respiratory syndrome coronavirus 2 (SARS-CoV-2) pandemic. Although the coronavirus disease 2019 (COVID-19) pandemic cannot in itself be credited for the radical change in healthcare that has been experienced over the last two years, there is no doubt that the digital transformation of healthcare has accelerated worldwide because of this pandemic.^2,3^

In talking about the change that healthcare is going through, from the analogue to the digital world, many people may think that certain simplistic actions (such as “computerizing the process”) will be enough to overcome the challenges imposed in this 21st century, in which we are increasingly digital beings. Perhaps they think that it will be enough to “digitize” healthcare as it is today and everything will be better, more beautiful and technological.

However, the process is not that simple. The patient healthcare system has developed centered on physical locations to which we are require to travel. All of this involves time: time to come or go; time to wait for care that never seems to arrive; and time to wait for the diagnosis. This time can often represent the patient’s chance of survival or death. In the current healthcare model, which is now outdated, we must go to where the infrastructure and professionals are, in order to receive diagnoses and care. Digital healthcare is now changing this.

When the internet first became available for the public to use, in the late 1990s, many opportunities to apply digital technologies to healthcare were considered. Some were more like science fiction (such as autonomous robotic telesurgery), and others we already see today as a reality of our daily care, such as telemedicine.^4^

To illustrate the role that the pandemic played in the adoption of telemedicine, many hospitals were able to benefit from teleconsultation platforms and teleconsultation services to remotely assist patients in ICUs, train multidisciplinary teams and thus fight COVID-19. The Instituto do Coração of Hospital das Clínicas, Faculdade de Medicina, Universidade de São Paulo (HCFMUSP) was one of the leaders in this movement in Brazil, having implemented a TeleICU service in record time to support several hospitals in the state of São Paulo that were on the front line of care for patients with COVID-19. The results so far have shown the great benefit of this telemedicine technological platform.^5^

New terms that were not part of our health vocabulary until recently, such as artificial intelligence, machine learning, virtual reality, big data, blockchain and wearables, among others, are revealing that we are now entering a completely new field, both for ordinary citizens and for medical professionals.

Another matter that needs to be considered is the change in the population’s culture due to the arrival of these new technologies. We have seen how our habits have changed through the popularization of mobile phones, starting in the 1990s, and very recently, through the new wave of digital habits that have arisen with the growth in smartphone use. We now have devices with which there is no longer any need to make traditional phone calls. Instead, internet-based interactive messaging apps can be used to record and send audio, texts or images. We keep on communicating, only differently!

The market for new wearables and remote diagnostic solutions is growing all the time and is expected to reach US$ 116 billion by 2025, according to studies by specialized companies.^6^ Today, this has become a new area of medicine and healthcare that is bringing in a revolution in the very way of understanding patients through the data that can be collected from them, thereby telling a much more trustworthy story of their health and habits.^7^

*Time* magazine featured the front-cover headline “Never Offline” in its September 11, 2014, edition.^8^ The article highlighted the global launch of the Apple watch (a watch in the smartwatch category). An editorial mentioned: “The Apple Watch is just the beginning. How wearable technology will change your life - like it or not.” After seven years, we can say they were right: we are now more connected than ever. We generate more data about ourselves than in all previous centuries put together. Today, with the technological resources available and those to come, we can monitor a huge variety of physiological, behavioral and emotional parameters of all human beings that are in some manner connected to this large digital data network that constitutes the internet.^9^ Nonetheless, are we aware of what is really happening?

Issues of individual data protection, invasion of privacy, cybersecurity, interoperability, inclusive digital culture for the population (so as not to generate a new society of digitally excluded people), etc., are on the agenda today.^10,11^ The implementation of digital healthcare is not only a matter of creating electronic clinical protocols, but also one of educating everyone involved: from physicians and healthcare professionals to patients and their caregivers. We are truly entering a digital social revolution.^12,13^

We are now born and are living “plugged into” the digital world. In healthcare terms, exabytes of data (big data) about our DNA, laboratory tests, clinical images and medical history, from electronic medical records registered in the blockchain, will increasingly become available. Data will also come from the wearables and smartphones that we use. All of these data will be analyzed through computers that make use of sophisticated artificial intelligence and exponential machine learning (machine learning and deep learning). Therefore, with regard to this essential issue of digital healthcare, which will determine much of our present and future, there is an absolute requirement for us to prepare ourselves for this today.

## References

[1] Daniel Stone (2017). The 10 Inventions that Changed the World.

[2] Petracca F, Ciani O, Cucciniello M, Tarricone R (2020). Harnessing Digital Health Technologies During and After the COVID-19 Pandemic: Context Matters. J Med Internet Res..

[3] Keesara S, Jonas A, Schulman K (2020). Covid-19 and Health Care’s Digital Revolution. N Engl J Med..

[4] Ohannessian R, Duong TA, Odone A (2020). Global Telemedicine Implementation and Integration Within Health Systems to Fight the COVID-19 Pandemic: A Call to Action. JMIR Public Health Surveill..

[5] Carvalho CRR, Scudeller PG, Rabello G, Gutierrez MA, Jatene FB (2020). Use of telemedicine to combat the COVID-19 pandemic in Brazil. Clinics (Sao Paulo).

[6] Global Wearables Market Anticipated to Reach US$116.88 Billion by 2025, Growing at a CAGR of 17% Between 2021 and 2025 - ResearchAndMarkets.com https://www.businesswire.com/news/home/20210930005681/en/Global-Wearables-Market-Anticipated-to-Reach-US116.88-Billion-by-2025-Growing-at-a-CAGRof-17-Between-2021-and-2025---ResearchAndMarkets.com.

[7] Piwek L, Ellis DA, Andrews S, Joinson A (2016). The Rise of Consumer Health Wearables: Promises and Barriers. PLoS Med..

[8] Lev Grossman, Matt Vella (2014). Never Offline.

[9] Rosser BA, Vowles KE, Keogh E, Eccleston C, Mountain GA (2009). Technologically-assisted behaviour change: a systematic review of studies of novel technologies for the management of chronic illness. J Telemed Telecare..

[10] Grande D, Luna Marti X, Feuerstein-Simon R (2020). Health Policy and Privacy Challenges Associated With Digital Technology. JAMA Netw Open..

[11] Gostin LO, Halabi SF, Wilson K (2018). Health Data and Privacy in the Digital Era. JAMA..

[12] Meskó B, Drobni Z, Bényei É, Gergely B, Győrffy Z (2017). Digital health is a cultural transformation of traditional healthcare. Mhealth..

[13] Birnbaum F, Lewis D, Rosen RK, Ranney ML (2015). Patient engagement and the design of digital health. Acad Emerg Med..

